# Evaluating the Modulation of the Acoustic Startle Reflex in Children and Adolescents *via* Vertical EOG and EEG: Sex, Age, and Behavioral Effects

**DOI:** 10.3389/fnins.2022.798667

**Published:** 2022-04-06

**Authors:** Anastasios E. Giannopoulos, Ioanna Zioga, Panos Papageorgiou, Panagiota Pervanidou, Gerasimos Makris, George P. Chrousos, Xanthi Stachtea, Christos Capsalis, Charalabos Papageorgiou

**Affiliations:** ^1^School of Electrical and Computer Engineering, National Technical University of Athens, Athens, Greece; ^2^Donders Institute for Brain, Cognition and Behavior, Radboud University Nijmegen, Nijmegen, Netherlands; ^3^First Department of Psychiatry, Eginition Hospital, National and Kapodistrian University of Athens Medical School, Athens, Greece; ^4^Department of Electrical and Computer Engineering, University of Patras, Patras, Greece; ^5^Unit of Developmental and Behavioral Pediatrics, First Department of Pediatrics, School of Medicine, “Aghia Sophia” Children’s Hospital, National and Kapodistrian University of Athens, Athens, Greece; ^6^Neurosciences and Precision Medicine Research Institute “COSTAS STEFANIS” (UMHRI), University Mental Health, Athens, Greece

**Keywords:** EEG, prepulse inhibition, prepulse facilitation, sensorimotor gating, acoustic startle reflex, first-derivative potential

## Abstract

Acoustic startle reflex (ASR) constitutes a reliable, cross-species indicator of sensorimotor and inhibitory mechanisms, showing distinct signature in cognitive aging, sex, and psychopathological characterization. ASR can be modulated by the prepulse inhibition (PPI) paradigm, which comprises the suppression of reactivity to a startling stimulus (pulse) following a weak prepulse (30- to 500-ms time difference), being widely linked to inhibitory capabilities of the sensorimotor system. If the prepulse–pulse tones are more clearly separated (500–2,000 ms), ASR amplitude is enhanced, termed as prepulse facilitation (PPF), reflecting sustained or selective attention. Our study aimed to investigate early-life sensorimotor sex/age differences using Electroencephalographic recordings to measure muscular and neural ASR in a healthy young population. Sixty-three children and adolescents aged 6.2–16.7 years (31 females) took part in the experiment. Neural ASR was assessed by two different analyses, namely, event-related potentials (ERPs) and first-derivative potentials (FDPs). As expected, PPF showed enhanced responses compared with PPI, as indicated by eyeblink, ERP and FDP measures, confirming the gating effect hypothesis. Sex-related differences were reflected in FDPs, with females showing higher ASR than males, suggesting increased levels of poststartle excitability. Intragroup age effects were evaluated *via* multipredictor regression models, noticing positive correlation between age versus eyeblink and ERP responses. Attention-related ERPs (N100 and P200) showed distinct patterns in PPI versus PPF, potentially indicative for alternative attentional allocation and block-out of sensory overload. Screening measures of participants’ neurodevelopmental (assessed by Wechsler Intelligence Scale for Children) and behavioral (assessed by Child Behavior Checklist) markers were also associated with increased N100/P200 responses, presumably indexing synergy between perceptual consistency, personality profiling, and inhibitory performance. Conclusively, modulation of ASR by PPI and PPF is associated with biological sex and internal/external personality traits in childhood and adolescence, potentially useful to guide symptomatology and prevention of psychopathology.

## Introduction

Human beings are tactically exposed to threatening events during their everyday activities (Barlow, 2000; McNaughton and Corr, 2004). Warning signals can prepare individuals’ motor and mental responses to sudden events *via* the firing of dedicated neural networks (Grillon, 2007). Those mechanisms are partially supported by the sensorimotor brain system (Swerdlow et al., 1999) and have been studied in the general frame of the “fight-or-flight” behaviors (Swerdlow et al., 2000). The acoustic startle reflex (ASR) refers to a coactivation of reflexive, involuntary responses triggered by a sudden, intense stimulus and has been linked to sensorimotor shaping and diverse forms of neuroplasticity (Davis et al., 1982; Braff et al., 2001). Modulation of ASR is increasingly being used for assessing cognitive and affective operations in healthy, clinical, and psychiatric groups (Braff et al., 2001). ASR is typically measured in terms of motor responses across all mammals, usually as an eyeblink response in humans (Swerdlow et al., 1999; Braff et al., 2001) and as a whole-body motor response in animals (Swerdlow et al., 2000). In the current study, we investigated the time course of ASR maturation, from young children to adolescent males and females, by evaluating electrophysiological responses to auditory startle stimuli. The potential effect of intelligence as well as personality and behavior characteristics was further examined. We thus adopted a tripartite approach by combining ocular, neural, and behavior measures to assess ASR between sexes and throughout youth.

Although ASR is an automatic reflex, its intensity can be modulated by habituation (ASR is reduced with habituation) (Ellwanger et al., 2003), fear (ASR increases with fear) (Davis et al., 1988), and prepulse–pulse pairing (Takahashi et al., 2011). Prepulse inhibition (PPI) is the normal ASR attenuation attributed to a weak nonstartling pulse (hereafter called as prepulse) shortly preceding (30–500 ms) an intense startling stimulus, whereas prepulse facilitation (PPF) refers to an ASR increase, occurring when the startling stimulus is lengthily preceded (500–2,000 ms) by the prepulse (Aasen et al., 2016; Stachtea et al., 2020). PPI has been systematically termed as the responsible oculomotor/neural phenomenon for inhibiting sensory overload by partially enabling sensorimotor gating mechanisms (Geyer et al., 1990). Neuronal circuitry underlying PPI combines the inhibition processing of incoming stimuli (i.e., startle), simultaneously with the an ongoing processing of a previously triggered stimuli (i.e., prepulse) (Naysmith et al., 2021). Although the mechanism underlying PPF is still unclear, it has been widely related to sustained attention (Aasen et al., 2016), attention orienting, and sensory enhancement (Naysmith et al., 2021), primarily as a result of excitatory neurons fired by both prepulse and pulse (gating effect is diminished due to the elongated prepulse–pulse interval). PPF is closely related to orienting (Wynn et al., 2005) and activation processes that involve passive attention to input (Siddle, 1991). Both mechanisms are thought to reflect protective function for reactively handling disruptions on the processing of prepulse signals, giving insights about not only low-level involuntary (automatic), but also high-level-controlled components of attentional modulation (Dawson et al., 1993; Li et al., 2009).

While PPI has been extensively studied in healthy adults (Braff et al., 2001), as well as neuropsychiatric (Wynn et al., 2005; Giannopoulos et al., 2021b) and clinical populations (Takahashi et al., 2011), the age groups of childhood and adolescence are relatively understudied. Most of the existing studies have reported PPI deficits in special prenatal conditions (Kponee-Shovein et al., 2020) and in pediatric disorders, such as Tourette syndrome (Buse et al., 2015), enuresis (Ornitz et al., 1992), and autism spectrum disorder (Takahashi et al., 2018). Maturation of startle modulation starts after the third year of life, with infants (2–6 months) and toddlers (15 months) (Balaban et al., 1989; Graham et al., 2015) not showing significant startle modulations. Development of the ASR has been also addressed in children, showing increasing maturation for the PPI between 3 and 10 years of life, reaching adult levels at 9–10 years (Gebhardt et al., 2012). Notably, previous studies that have shown full maturity of the ASR reflex at approximately 8 years old have not included older children in their studies (Ornitz et al., 1986, 1991). Extended evidence posits that PPI/PPF constitute reliable biomarkers, being continuously linked with clinical severity, high-risk illnesses, cognitive impairments, psychiatric disorders, and social dysfunction (Siddle, 1991; Dawson et al., 1993; Wynn et al., 2005; Li et al., 2009; Kponee-Shovein et al., 2019; Jafari et al., 2020; Giannopoulos et al., 2021b). As PPI/PPF have stable theoretical and experimental documentation, their investigation in childhood/adolescence could indicate early development of disorders. Furthermore, joint consideration of ASR modulation and other clinical or screening measures would allow the early identification and prolepsis of deficient neural function in high-risk clinical groups (Naysmith et al., 2021).

With regard to sex effects on startle reflex responses, related work on adult and preadult populations has demonstrated sexual dimorphism (Kumari et al., 2004, 2005; Aasen et al., 2016). Specifically, women exhibit weaker inhibition than men (Kumari et al., 2004; Aasen et al., 2016), even with the absence of confounding factors, such as personality traits and smoking (Aasen et al., 2016; Naysmith et al., 2021). Women’s lesser inhibition of the ASR in PPI has been attributed to reproductive hormones related to estrogen and dopaminergic activity (Aasen et al., 2016; Naysmith et al., 2021), whereas the early follicular stage of the menstrual cycle has been associated with enhanced PPI than the luteal phase (Kumari et al., 2010). PPF is generally higher in females than males in adults and pre-adults, with prepubescent girls (8+ years) showing higher PPF than prepubescent boys (Ornitz et al., 1991; Kumari et al., 2003, 2010). Neuroscientific studies have also suggested weaker P50 gating in healthy females compared with males (Hetrick et al., 1996; Patterson et al., 2008).

Other studies have shown influences of personality trait–emotionality in PPI, with high anxiety increasing the eyeblink responses and low fear levels indicating enhanced N100/P200 amplitudes (De Pascalis et al., 2013). Anxiety and depression have also been associated with enhanced late positive evoked potentials in response to emotional stimuli (Macnamara et al., 2016; McLean et al., 2020) and negatively valenced words (Maki et al., 2002; Jovanovic et al., 2004). Attention biases for negative stimuli in depressed children and adolescents have been widely demonstrated (Stewart et al., 2014). On the other hand, higher intelligence and better attention abilities are associated with neurophysiological efficiency, as demonstrated in studies with adults (Deary and Caryl, 1997) as well as children with attention-deficit/hyperactivity disorder (ADHD) (Mayes et al., 1998). Sociodemographic predictors of PPI in children and adolescents have been also used to identify early children at risk for neurodevelopmental disorders (Kponee-Shovein et al., 2019; Naysmith et al., 2021).

To our current knowledge, the existing PPI literature is limited with regard to healthy young populations, and direct comparisons between PPI and PPF responses are rare. Also, joint evaluation of ocular and neural PPI/PPF is understudied (e.g., see San-Martin et al., 2018 for adult population), with the majority of studies reporting only muscular ASRs. Here, we jointly evaluated ocular and neural ASR in children and adolescents (aged 6–17 years) through a PPI and PPF paradigm. Key objectives of the study included the elucidation of whether biological sex affects oculoelectrophysiological ASR and whether maturation of the ASR is observed in these measures in a young healthy sample. We further aimed to examine potential correlations between ocular/neural ASR responses and behavioral measures and general perceptual skills. Specifically, two psychometric scales were obtained to assess participants’ psychological and behavioral profile, namely, the Wechsler Intelligence Scale for Children [WISC-III, (Wechsler and Kodama, 1949)] and a short form of the Child Behavior Checklist [CBCL, (Achenbach and Edelbrock, 1991)]. Both tools have been widely used to assess neurocognitive profiles and to rate behavioral problems and skills, respectively, and are partially co-established as *Diagnostic and Statistical Manual of Mental Disorders* prognostic criteria (Krol et al., 2006; Regier et al., 2013).

With regard to the oculographic analysis, we followed the conventional scheme for evaluating the vertical eyeblinks, and we outlined a classification method for categorizing “reflexive” and “nonreflexive” eyeblink-related ASRs based on widely used rejection criteria (Blumenthal et al., 2005). In the electrophysiological analysis, we used the typical event-related potential (ERP) technique (Kappenman and Luck, 2016), as well as a new assessment technique based on the first-order derivation (Majumdar, 2012; Liu and Lin, 2019) of ERPs [first-derivative potentials (FDPs)]. FDP analysis can uniquely describe the neural responses (Majumdar, 2012), focusing on the whole-scalp (WS) electroencephalographic (EEG) parts where abrupt signal changes are observed (as commonly seen in poststartle responses) and eliminate baseline drifts (Liu and Lin, 2019). The FDP technique thus combines the temporal specificity of EEG (as seen in ERPs) with a baseline-robust approach, useful for identifying global (widespread) evoked responses. Associations between sex/age/behavioral scales and ocular/EEG measures were tested *via* multipredictor regression models.

Driven by the functional significance of PPI/PPF mechanisms in the human sensory system, this study aims to quantify some of the electrophysiological alterations underlying child’s and adolescent’s sensorimotor inhibition, potentially to be used as complementary prediagnostic alerts and early signs of neural dysfunction. Based on the aforementioned studies, our first hypothesis was that PPI would show weaker poststartle ocular and neural responses compared with PPF, due to sensory gating in the former condition. Based on previous research (Ornitz et al., 1991; Kumari et al., 2004; Aasen et al., 2016), females were expected to show enhanced PPI than males, although there are arguments for the opposite (Hetrick et al., 1996; Patterson et al., 2008) or no sex effects (Freedman et al., 1987; Waldo et al., 1987). In any case, PPI sex differences are understudied in young ages, and we expected PPI enhancements in females reflecting increased poststartle excitability (Kofler et al., 2013) due to unstable hormonal states. Furthermore, we hypothesized that PPI and PPF responses would be correlated with age, providing evidence for maturation of the ASR throughout aging in youth. In line with previous literature on internalizing problems and alertness (Beck, 1976), we expected that high internalizing would be associated with impaired PPI responses, due to decreased suppression of threatening stimuli in individuals with high anxiety and depression. We also explored potential effects of externalizing on startle modulation responses. Finally, we hypothesized that intelligence would be positively correlated with successful sensorimotor gating, thus weaker responses to PPI and/or stronger responses to PPF.

## Materials and Methods

### Participants

Sixty-six school-aged children/adolescents and their parents/caregivers participated in the experiment. Participants were recruited from a convenience community sample of children from the area of Athens. Finally, 63 were included in the data analyses of this work, as three participants were excluded as “nonresponders” in the ASR of eyeblinks (see *Startle Eyeblink Response to Acoustic Stimuli*). The age of the children ranged from 6.17 to 16.67 years, whereas the biological sex was balanced (32 males and 31 females). Males were aged 10.59 ± 2.19 years, and females were aged 11.04 ± 2.49. No significant age differences were observed between the two groups [*t*(61) = −0.761,*p* = 0.450]. Written consent was obtained from all participants’ parents, after providing a detailed description of the experimental procedure. Inclusion criteria comprised healthy children and adolescents, 6–17 years old of both sexes, and ability to speak and write in Greek. Children with chronic physical illnesses, genetic/chromosomal disorders, intellectual disability, psychiatric disorders, and auditory/vision problems were excluded from the study. During the recording of the medical history, the participants’ parents (or caregivers) confirmed the absence of auditory/visual impairments, smoking, and substance abuse. Exclusion criteria were met when the CBCL total score exceeded the value of 60 (Achenbach, 1991; Achenbach et al., 2011), as an indication of borderline or clinical marker (see *Intelligence Measures and Behavioral Ratings* for details on CBCL ratings). Participants provided all the necessary demographic information, before progressing to the screening and assessment procedures. All necessary clinical measurements were conducted by registered clinicians and parents–caregivers of the sample.

The study was performed in the psychophysiology laboratory of the University Mental Health, Neurosciences and Precision Medicine Research Institute “Costas Stefanis” (U.M.H.R.I.), in collaboration with the First Department of Psychiatry, Medical School, Eginition Hospital, National and Kapodistrian University of Athens and the Unit of Developmental and Behavioral Pediatrics, First Department of Pediatrics, School of Medicine, National and Kapodistrian University of Athens, Athens, Greece. The study was conducted according to the guidelines of the Declaration of Helsinki, and was approved by the Scientific and Ethics Committee of “Aghia Sophia” and “Aiginiteion” Hospitals (protocol no. 298/01-06-2016).

### Intelligence Measures and Behavioral Ratings

Two measures were obtained from each child prior to their participation in the study. Specifically, the WISC-III was used to assess cognitive abilities in discrete domains (Wechsler and Kodama, 1949). A total WISC score, namely, the Full-Scale IQ, represented children’s general intellectual ability. To further identify behavior problems, the CBCL was also completed by the children’s parents–caregivers (Achenbach and Edelbrock, 1991). The total problems score comprised individual scales such as aggressive behavior, anxious/depressed, attention problems, rule-breaking behavior, somatic complaints, social problems, thought problems, and withdrawn/depressed subscales.

#### WISC-III

The Greek-translated version of WISC-III test (Georgas et al., 1997) was acquired to assess individual and global intelligence markers for children aged between 6 and 16 years (plus 11 months). Four intelligence disciplines were evaluated to index child neurodevelopmental abilities, using a short form of WISC-III. This 4-item form constitutes a time-efficient compromise between psychometric–clinical and practical variables with reliability greater than 0.9 (Kaufman et al., 1996). Verbal, perceptual, practical, and arithmetical abilities were indexed by the following scales:

• *Similarities:* It belongs to the verbal subscales and evaluates the ability of the child/adolescent for logical abstract thinking. More specifically, the examinee is asked to find the conceptual correlation/classification that exists between 2 words (e.g., “What does the piano and the guitar look like? Anger and joy, ice and steam”).
• *Block design:* It belongs to the practical subscales and evaluates the ability to solve nonverbal problems using visual perception and perceptual organization. The examinee is asked to construct 12 designs using dichromic cubes. The level of visual–motor coordination, composition of a wholeness from individual pieces, and spatial perception are also significantly controlled.
• *Arithmetic:* This arithmetic subscale belongs to the verbal subscales and assesses not only the ability for simple mathematical calculations, but also verbal comprehension. The child is asked to solve arithmetic problems without pen or paper. The child’s ability to concentrate and think under the pressure of time is also taken into account.
• *Picture completion:* This is a practical subscale for evaluating the speed of visual recognition/perception of specific stimuli. The child is asked to detect the missing part of colored images showing common objects.

#### CBCL/4-18

CBCL is a screening checklist for evaluating children’s subfields of behavior (Achenbach, 1991). The Greek version of CLBL (Roussos et al., 1999) was also obtained from children’s parents, appropriate for screening social behavior, attention, and aggressiveness in children aged from 6 to 18 years (Motti-Stefanidi et al., 1993). Given our experimental task related to attention, the following three general screening measures were extracted from the complete CBCL form:

• *Attention problems:* This scale includes 11 items such as “can’t sit still,” “daydreams,” and “can’t concentrate,” including features of inattention, hyperactivity, and impulsivity. All items were scored on a three-point scale, reflecting the occurrence of behavioral problems during the preceding 6 months (0 if the item was not true, 1 if the item was somewhat or sometimes true, and 2 if the item was very true or often true).
• *Internalizing:* It is a broad-band scale that sums anxious/depressed, withdrawn-depressed, and somatic complaints subscores.
• *Externalizing:* It is also a broad-band metric that accumulates rule-breaking and aggressive behavior.

### Electroencephalographic Acquisition

**FIGURE 1:**
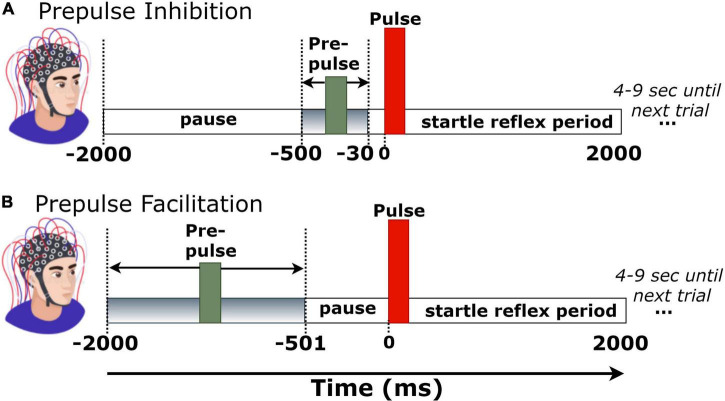
Trial structure of **(A)** prepulse inhibition (PPI) and **(B)** prepulse facilitation (PPF) conditions.

Electroencephalographic measurements were captured in a Faraday cage. Before the session began, participants sat in a comfortable chair and were asked to hold their heads firmly fixed during the session recordings. They were instructed to move their heads for resting purposes throughout the intertrial interval (4- to 9-s resting period, see also Figure 1). Five testing trials were performed to familiarize individuals with the experimental conditions. Participants’ electrophysiological activity was digitalized at a sampling frequency of 1,000 Hz from an elastic cap (procured by Electro-Cap Center B.V.) with 30 uniformly placed (active) electrodes. The electrode placement was in accordance with the International 10–20 System following the labeling defined in Modified Combinatorial Nomenclature. Specifically, the electrode composition was {FP1/FP2, FPZ, AFZ, F3/F4, F7/F8, FZ, FC3/FC4, FCZ, FT7/FT8, CZ, T7/T8, CP3/CP4, CPZ, TP7/TP8, P3/P4, P7/P8, PZ, O1/O2, OZ}. The brain signals were amplified by a Braintronics DIFF/ISO-1032 amplifier and then driven to an input of a National Instruments PCI-6255 DAQ card (16 bits analog-to-digital conversion). Electrode impedance was kept constantly below 5 kΩ during acquisition. EEG online activity was referenced to the average of earlobes, whereas the ground electrode was mounted on the left mastoid. Eye movements were also recorded by two electrodes placed above and below the left eye and another two on the outer canthi. This placement gave two bipolar signals for each child, namely, the vertical electro-oculogram (VEOG, as the difference between the upper and lower EOG channels) and horizontal electro-oculogram (HEOG, as the difference between the left and right EOG channels).

### Paradigm and Trial Structure

Each experimental block consisted of 51 pairs of tones. Fifty-one pairs of tones consisted a single-trial block. The first tone (i.e., prepulse) had an intensity of 60 dB, whereas the second tone (pulse or startle) was at 100 dB. To verify the expected output intensity of the two pulses (60 and 100 dB), the intensities of both stimuli were measured and calibrated *via* a sound level meter prior to the experimental sessions. Stimuli were presented auditorily *via* headphones in both left and right ears. During the experimental session, there were 25 trials with long prepulse–pulse time intervals (501–2,000 ms), corresponding to the PPF condition, and 26 trials with short prepulse–pulse intervals (30–500 ms), corresponding to the PPI condition. To avoid habituation, presentation order of trials was pseudorandom across participants, whereas the prepulse–pulse temporal separation was randomly set at 30–500 ms (for PPI) or 500–2,000 ms (for PPF) in each trial (Giannopoulos et al., 2021b). All tones had a duration of 40 ms, a frequency of 2,000 Hz, and rise/fall times of 0.5 ms. Each trial recording had a duration of 4 s (–2 to 2 s, time-locked to startle tone onset). The intertrial interval varied randomly between 4 and 9 s, to avoid prediction effects of stimulus presentation. Figure 1 depicts the trial structure in PPI and PPF conditions.

### Preprocessing

Electroencephalographic datasets were preprocessed using the EEGLAB (version 2019.1) toolbox (Delorme and Makeig, 2004). First, EEG data were downsampled at 250 Hz (1 sample per 4 ms) to compress the data sizes. Then, a digital high-pass filter at 1 Hz was applied, followed by a notch filter with a stop-band of 45–55 Hz to suppress line noise. Both filters were implemented using the EEGLAB’s filter function (*pop_eegfiltnew.m*), whose implementation relies on a zero-phase Hamming-windowed sinc finite-impulse response filter parameterized as follows: transition bandwidth = 1 Hz, filter length = 827, roll-off = –6 dB/octave (Widmann et al., 2015). Electrodes showing abnormal time course (flat or extreme values) were identified using the *clean_artifacts.m* function and interpolated based on neighboring channels. The activity of each channel was then re-referenced to the WS common average.

Independent component analysis (ICA) was then performed to decompose the signals into independent activations, especially focusing on the blink-contaminated components. The SASICA plug-in tool (Chaumon et al., 2015) was used to semi-automatically guide the annotation of non-brain (blinks and/or saccades) components. Component rejection criteria included simultaneous consideration of “autocorrelation” (weak autocorrelation reflects noisy components), “focal components” (bad channels have too focal components), “focal trial activity” (components with focal trial activity correspond to non-brain ones), “EOG correlation” (blink and saccade components are correlated with VEOG/HEOG), “ADJUST” (Mognon et al., 2011), and “FASTER” (Nolan et al., 2010) methods. As the blink-related artifactual components were spatially localized at frontal areas, channels FP1/FP2/FPZ were excluded from the analyses. The mean number of channels (per participant) was 25.2 ± 1.1. Continuous and artifact-free EEG data were then segmented into 4-s epochs (–2 to 2 s), time-locked to startle-tone onset. Finally, a baseline correction was performed by subtracting the mean of –30- to 0-ms prestartle activity in each trial. This narrow baseline ensured no overlaps with the prepulse tone of PPI trials (Figure 1A).

### Data and Statistical Analyses

#### Startle Eyeblink Response to Acoustic Stimuli

In the first part of the analyses, the ASR was evaluated from the ocular responses. The blink-related signal, namely, the VEOG, was processed for each individual participant following the technical instructions mentioned in (Blumenthal et al., 2005). The goal of this standardized approach was to maximize the signal-to-noise ratio (usually low in VEOGs) and detect actual blink responses that can be discriminated from the non–startle-related blinks. First, VEOG signals were bandpass filtered at 28–300 Hz, as the typical power spectra of acoustically elicited eyeblink (Blumenthal et al., 2005; San-Martin et al., 2018). This filtering configuration cancels out low-frequency artifacts caused by eye stretching, overlapping electrode collars, retinal potentials, and other facial muscle activity, as well as by high-frequency artifacts associated with instrumentation noise and electromagnetic interference of power line harmonics (van Boxtel, 2010). Filtering was performed *via* an infinite impulse response (fourth-order Butterworth filter, roll-off 24 dB per octave) (van Boxtel et al., 1998). Then, the VEOG signals were rectified, and the analytic peak envelope of the rectified signals was extracted for smoothing purposes.

As ASR blinks are the response of interest, spontaneous blinks constitute task-irrelevant artifacts. A proper trial rejection strategy was adopted (Blumenthal et al., 2005), discriminating the trials between “reflexive” and “nonreflexive” blinks, primarily based on two criteria: (i) reflexive trials show peak startle reflex in the typical latency window of acoustically elicited blinks (20–150 ms after startle), and (ii) reflexive trials do not include blinks occurring immediately before startle onset, even if they are similar in amplitude to startle blink responses. To test whether a single trial met these criteria, we *Z*-scored each individual trial based on a narrow prestartle period and tested whether the startle reflex window shows reduced blinking. Specifically, the VEOG single-trial signals were first *Z*-scored according to the following formula:


(1) $${\text{Z}}_{t}=\frac{{\text{VEOG}}_{t}-\text{MEAN}\left\{{\text{VEOG}}_{t=-30}^{t=0}\right\}}{\text{SD}\left\{{\text{VEOG}}_{t=-30}^{t=0}\right\}}$$

where *t*ϵ[−1,940, 2,040] ms, *Z_t_* is the *Z* score at time *t*, *VEOG_t_* is the non–*Z*-scored VEOG value at time *t*, and $\text{MEAN}\left\{{\text{VEOG}}_{t=-30}^{t=0}\right\}$ and $\text{SD}\left\{{\text{VEOG}}_{t=-=-30}^{t=0}\right\}$ are the mean and standard deviation (SD) across the prestartle (–30 to 0 ms) VEOG responses, respectively. This *Z*-transformation reflects how many SDs above the prestartle period are the VEOG response. Finally, the trials were labeled as “reflexive” if and only if the poststartle mean reflex within 20–150 ms was greater than 1; otherwise, they were labeled as “nonreflexive.” By doing so, it was ensured that the valid/reflexive trials are those corresponding to high early blinks relative to the prestartle period.

**FIGURE 2:**
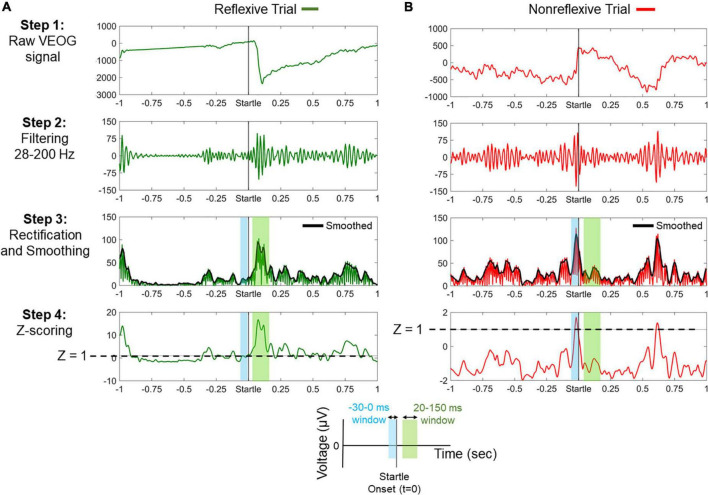
Processing steps for the classification between reflexive and nonreflexive trials based on the vertical electro-oculogram (VEOG) signal. **(A)** An example of a reflexive trial showing a strong blink immediately after startle onset. In step 4, it is shown that the poststartle *Z* score (within 20–150 ms) exceeds the threshold of 1 (dashed line), meaning that the blink is at least 1 SD above the prestartle –30- to 0-ms period. **(B)** An example of a nonreflexive trial showing the presence of a strong blink immediately before startle onset and a non–startle-related blink approximately 600 ms. In step 4, negative values of the poststartle *Z* scores (within 20–150 ms) indicate that the prestartle blink was dominant. Color-shaded areas highlight the prestartle (blue) and poststartle (green) windows of interest, as indicated by the legend at the bottom.

Figure 2 illustrates the processing steps for the classification of trials in “reflexive” and “nonreflexive” and the extraction of VEOG amplitude and latency. For instance, a valid blink can be observed within the window 20–150 ms, whereas a nonreflexive trial could include a strong blink immediately before startle onset. Note that a blink occurring outside the 20- to 150-ms range (such that of Figure 2B, approximately 600 ms) is considered non–startle-related.

As mentioned in *Participants*, three participants were excluded because of their low number of responsive trials (<18), whereas the rest of the participants showed at least 20 responsive trials. There was also no significant difference between males (responsive trials 22.78 ± 1.39 in PPI; 22.72 ± 1.63 in PPF) and females (22.65 ± 1.25 in PPI; 22.26 ± 1.41 in PPF) in the number of responsive trials [PPI: *t*(61) = 0.41,*p* = 0.68; PPF: *t*(61) = 1.19,*p* = 0.24]. The mean amplitude and peak latency were detected from each VEOG channel within the poststartle window at 20–150 ms. Those measures were extracted for each individual trial, and the single-subject measure was derived by averaging across trials (Blumenthal et al., 2005; van Boxtel, 2010).

Separately for VEOG amplitudes and latencies, a mixed-model 2 × 2 analysis of variance (ANOVA) with *condition* (2 levels: PPI vs. PPF) as the within-subjects factor and *group* (2 levels: males vs. females) as the between-subjects factor and/or their interaction effects were conducted to assess ocular ASR responses. Paired-samples *t* tests were conducted to compare the conditions (PPI vs. PPF) in the case of significant main effect of condition. ANOVA test statistics were corrected using Greenhouse-Geisser adjustments to avoid sphericity violations, whereas the *p* values of *post hoc* tests were Bonferroni-corrected. Between-groups equality of variance was verified by Levene tests (*p*′s = 0.05 for both conditions). All statistical procedures were performed using SPSS and MATLAB software, following the statistical thresholds of α = 5%.

#### Acoustic Startle Reflex as Neural Electroencephalographic Response

**FIGURE 3:**
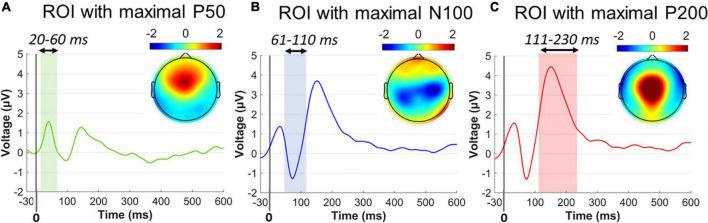
Collapsed ERP waveforms in three ROIs during –30 to 600 ms. **(A)** ERP wave for the P50 ROI and the P50 topographical distribution averaged across 20–60 ms poststartle. **(B)** ERP wave for the N100 ROI and the N100 topographical distribution averaged across 61–110 ms poststartle. **(C)** ERP wave for the P200 ROI and the P200 topographical distribution averaged across 111–230 ms poststartle. Color-shaded areas indicated the window elicitation of the aforementioned ERP components. Time 0 corresponds to the startle onset.

**FIGURE 4:**
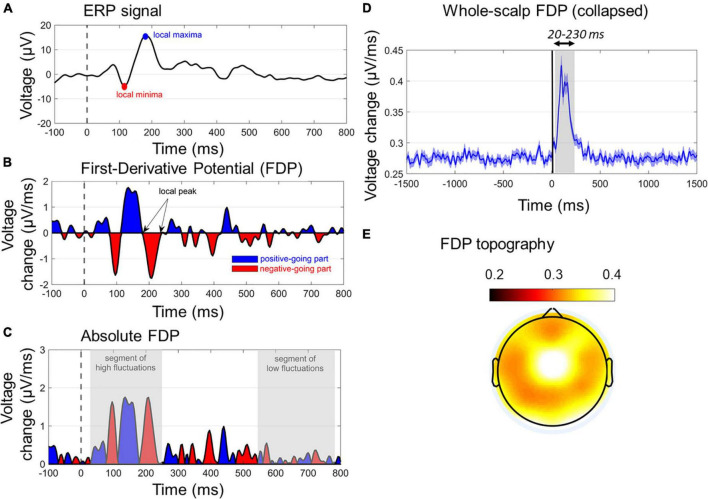
Characteristics of first-derivative potentials (FDPs). **(A)** A typical ERP wave with two dominant early peaks. **(B)** The respective FDP curve of ERP, indicating the peaks (zero values), positive-going (blue segments), and negative-going (red segments) of ERP. The temporal differentiation has been derived with Δ*t* = 4 ms. **(C)** The absolute FDP curve, highlighting the segments of rapid (early gray-shaded area) and slow (late gray-shaded area) in ERP waveform. **(D)** The whole-scalp FDP averaged across subjects and conditions. The window of FDP burst (20–230 ms) is also gray-shaded. **(E)** The topographical map of the grand-average FDP within the window of 20–230 ms.

The ASR-related electrophysiological responses were also evaluated following two signal processing methods: (i) the conventional ERP analysis (Kappenman and Luck, 2016) (Figure 3) and (ii) an alternative approach that is based on the first-order derivatives of the ERP signals (FDP) (Figure 4). ERP analysis allowed us to evaluate the spatiotemporal ASR responses, offering good temporal precision of the ASR-elicited ERP components. FDP analysis is also adopted to contrast the ASR responses across groups/conditions, giving insights about the extent of which abrupt ERP changes actually occur after the startle onset (Majumdar, 2012). FDP analysis pipeline results into a baseline-independent (Liu and Lin, 2019) and WS electrophysiological index, especially suited for a unique characterization of ASR. Time windows were selected based on the “collapsed localizer” technique (Luck and Gaspelin, 2017), which involves averaging across all groups and conditions to visually inspect the temporal segments/locations of general task engagement.

##### ERPs

Single-subject ERP waveforms were conventionally extracted by averaging across trials, separately for PPI and PPF conditions. Three dominant poststartle (early) ERP components were observed from the collapsed ERPs (i.e., grand-averages across subjects and conditions), namely, the positive-going P50 (20–60 ms, frontocental), the negative-going N100 (61–110 ms, central), and the positive-going P200 (111–230 ms, centroparietal) components. Allof these components have been systematically observed in PPI/PPF paradigms (Patterson et al., 2008; De Pascalis et al., 2013; Giannopoulos et al., 2021a,b), mainly associated with sensorimotor gating, attentional allocation, and orientation cognitive processes in responses to the ASR. For visual inspection purposes, Figure 3 illustrates the collapsed ERPs averaged across three regions of interest (ROIs), where each component was primarily distributed: ROI for P50 = {AFZ, FZ, F3/F4, FCZ, FC3/FC4}, ROI for N100 = {FCZ, FC3/FC4, CZ, C3/C4, CPZ, CP3/CP4}, ROI for P200 = {FCZ, CZ, C3/C4, CPZ}.

To compare ERP responses (amplitudes and latencies) between groups/conditions, a component-specific mixed 2 (males vs. females) × 2 (PPI vs. PPF) ANOVA was conducted with identical statistical corrections adopted for VEOG (see *Startle Eyeblink Response to Acoustic Stimuli*). For each component’s window and ROI, the mean amplitude (across timepoints) and peak latency were extracted as the single-subject dependent variables. Note that, as we test for three components, the *p* values for considering a main effect or interaction as significant were set to 0.05/3 = 0.167 in order to reduce the sensitivity in identifying statistically significant effects (type I errors).

##### First-Derivative Potentials

Apart from the ERP analysis, an alternative method is proposed for studying the ASR neural responses, based on the first-order differentiation across the timepoints of ERPs (Majumdar, 2012). Computationally, FDPs are derived by subtracting neighboring timepoints (separated by Δ*t*) of the ERP curve, according to the formula:


(2) $${\text{F}\text{D}\text{P}}_{n,t}=\frac{\partial }{\partial \text{t}}\left({\text{E}\text{R}\text{P}}_{n,t}\right)=\frac{{\text{E}\text{R}\text{P}}_{n,t+\Delta t}-{\text{E}\text{R}\text{P}}_{n,t}}{\Delta \text{t}}$$

where FDP_n,t_ and ERP_n,t_ stand for FDP and ERP, respectively, at time *t* and channel *n*, whereas Δ*t* notifies the differentiation step. FDPs are expressed in units of μV/ms, when ERPs are in μV, and the time scale Δ*t* is in the order of ms. As implied in Eq. (Barlow, 2000), FDP tracks the rate of change of the ERP waves, rather than reflecting the actual voltage values of the scalp activity. The definition of FDP enables several benefits in the signal processing pipeline, primarily including the following:

(i) Elimination of baseline drifts that are strong in ASR-recorded signals, mainly because the prepulse response affects the prestartle baseline period. To ensure high suppression of baseline drifts, the lowest Δ*t* can be selected (here Δ*t* = 1/250Hz = 4ms). This also implies that the need for baseline subtraction in FDP calculations is eliminated.
(ii) Suitability for capturing the signal segments with abrupt changes, rather than focusing on slow (and usually late) ERP components with gradual increases/decreases. This is especially beneficial in PPI/PPF paradigms, where the ASR-elicited components are early (< 300 ms) showing rapid fluctuations.
(iii) Sensitivity in identifying the peaks, the positive- and negative-going parts of the ERP waves. This is contradictive to the classical approach of calculating the mean amplitude e.g., for N100, which can be positive-valued but negative-going. In this sense, FDP can precisely reflect the slope of the ERP curve (positive/negative FDP corresponds to positive-/negative-going ERP, respectively, and zero FDP corresponds to ERP peak).
(iv) Time-window selectivity based on the temporal frames during which FDP bursts are observed (Figure 4D).
(v) Extension of the information provided by ERP in a denoised manner, as FDPs maintain the when and where of ERP peaks by further filtering out background slow oscillations.

Figures 4A–C show the main characteristics of an FDP derived by a single-channel ERP. In Figure 4C, the absolute FDP values have been calculated to focus on the unsigned rates of change.

##### FDP as WS-FDP

As absolute FDP represents the rate of change of the ERP responses, here it was used to uniquely quantify the ASR in a single-measure WS manner. This was done by taking the mean FDP (timepoint-wise) across all channels (except FP1/FP2/FPZ due to potential blink contaminations). Note that keeping the absolute values of FDP before calculating the WS-FDP is performed to avoid mutual cancelations between negative and positive-valued segments of signed FDP waves. WS-FDP reflects the unsigned time evolution in the average WS rate of change in μV/ms. Figure 4D shows the collapsed WS-FDP time course, highlighting the area of excessive rate of change (20–230 ms) and restricting the prestartle and late signal parts of smooth changes. This is consistent with ERP components time windows, spanning from P50 onset to P200 offset. Finally, Figure 4E shows the topographical map of FDPs within the 20–230 ms poststartle. In a general point of view, WS-FDP isolates the segment of rapid changes in the shape of the WS ERPs, eliminating the task-irrelevant parts of the signal, as indicated by Figure 4D (prestartle and > 250-ms activity oscillates approximately 0.27 μV/ms).

Separately for PPI and PPF, single-subject FDP measures were detected by computing the mean amplitude and peak latency in the 20- to 230-ms time window of the respective WS-FDP curve. To avoid intersubject variability in the prestartle period, WS-FDPs were baselined (subtraction of mean across -30 to 0 ms) before the calculation of FDP measures. Similarly (see *Startle Eyeblink Response to Acoustic Stimuli*), ANOVA and *post hoc* testing was carried out to examine group/condition effects on the WS-FDP scales.

### Startle Reflex, Age, and Behavior Associations

We tested potential correlations between electrophysiological ASR measures and sex, age, and behavioral scores (WISC-III and CBCL). Stepwise multiple linear regression (SMLR) models (Henderson and Denison, 1989) were conducted considering the categorical sex, scalar age, and WISC-III and CBCL scores as dependent variables and the VEOG/EEG measures as predictors. Models contained an intercept, linear terms for each predictor, and all products of pairs of distinct predictors (no squared terms). The SMLR model construction relies on adding and removing terms from a linear or generalized linear model based on their statistical significance in explaining the response variable. Significant predictors were those corresponding to *p* < 0.05, when the null hypothesis was “If the predictor will be added to model, it will have zero coefficient.”

## Results

### Behavioral Analysis

In the behavioral level, screening measures of perceptual skills and behavioral problems were contrasted between males and females. Table 1 summarizes the statistics of the group mean WISC-III and CBCL metrics. Evidently, there were no sex differences in the screening measures, except for WISC-III “Similarities,” with males scoring higher than females. The absence of significant differences ensures that no confounding factors in between-groups comparisons are presented in terms of WISC-III and CBCL performance. There were also no correlations between age and WISC or age and CBCL scores (*R* coefficients range = –0.2 to +0.2).

**TABLE 1:** Behavioral data comparisons between groups (males vs. females).

Measure	Subscale	Males		Females		Statistics	
		Mean	SE	Mean	SE	*t* value	*p* value
WISC-III	Similarities	16.31	0.47	14.81	0.36	*t*(61) = 2.54	0.01*
	Block design	13.28	0.56	13.06	0.38	*t*(61) = 0.32	0.75
	Arithmetic	13.72	0.50	12.58	0.46	*t*(61) = 1.68	0.10
	Picture completion	9.50	0.54	10.16	0.45	*t*(61) = –0.94	0.35
	Full-scale IQ	120.72	2.58	116.94	1.92	*t*(61) = 1.17	0.25
CBCL	Attention problems	51.53	0.55	52.61	0.68	*t*(61) = –1.24	0.22
	Internalizing	48.25	1.50	46.07	1.57	*t*(61) = 1.01	0.31
	Externalizing	45.50	1.47	48.38	1.26	*t*(61) = –1.49	0.14
	Total problems	46.10	1.40	46.10	1.30	*t*(61) = = 0.01	0.99

SE, standard error of the mean. *Statistically significant difference.

Confirmatively, to investigate whether the aggressiveness- and anxiety-related CBCL differences between males and females can be revealed in a more detailed age specificity, we repeated the comparisons in the subgroups of 6.2–12.9 and 13–16.7 years (all *p*’s > 0.05). However, those contrasts are characterized by low sample sizes, given the further splitting of the study population.

### Ocular Responses

**FIGURE 5:**
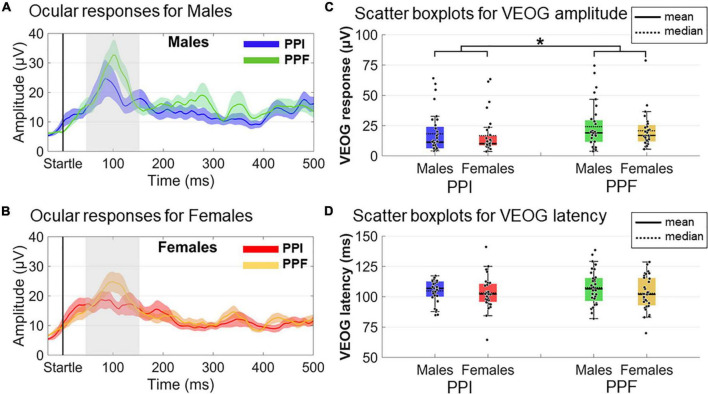
Grand-averaged VEOG responses for each group (males vs. females) and condition (prepulse inhibition, PPI, vs. prepulse facilitation, PPF). In panels **(A,B)**, the time course of the VEOG responses is illustrated for males (PPI: blue, PPF: green) and females (PPI: red, PPF: orange), respectively, from –30 to 500 ms (time 0 is the startle onset). Color-shaded areas correspond to the time course of the standard error (± 1 SE) of the mean. In panels **(C,D)**, the individual samples and descriptive statistics (mean and median) are depicted in scatter boxplots, separately for amplitudes and latencies, respectively. *Significance at *p* < 0.05.

VEOG measures (amplitude and latency) within the 20- to 150-ms poststartle range were used to assess the ocular blink reflexes (Figure 5). A 2 (sex: males, females)×2 (condition: PPI, PPF) mixed ANOVA revealed a significant main effect of condition ($F\left(1,61\right)=6.421;p=0.014;\eta_{p}^{2}=0.095$), with PPF (M ± SE = 22.55 ± 2.04 μ V) exhibiting overall higher amplitude than PPI (M ± SE = 17.53 ± 2.02 μ V). There was no significant effect of group (*p* = 0.378) or interaction (*p* = 0.643) between the variables. No remarkable effects were observed for VEOG latencies (all *p*’s > 0.25), with all peak latencies being identified approximately 100–110 ms of the poststartle period.

### Event-Related Potentials

**FIGURE 6:**
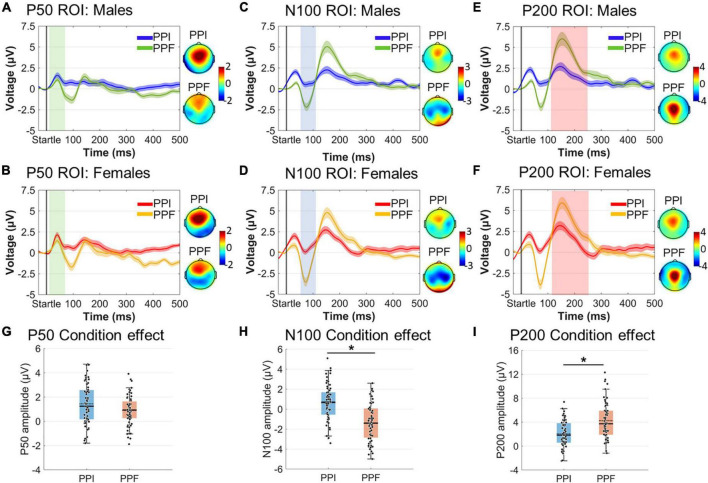
Grand-averaged ERP responses for each group (males vs. females) and condition (PPI vs. PPF). **(A–F)** The time course of the component- and ROI-specific ERP responses are illustrated for males (PPI: blue, PPF: green) and females (PPI: red, PPF: orange), respectively, from –30 to 500 ms (time 0 is the startle onset). Color-shaded areas correspond to the time course of the standard error (± 1 SE) of the mean. ERP component topographies are shown on the right of each plot. **(G–I)** The descriptive statistics (mean and median) of condition main effects are depicted as scatter boxplots for each ERP component. *Significant differences between PPF and PPI.

Amplitude and latency effects concerning spatiotemporally defined ERP components were evaluated *via* 2 (sex: males, females) × 2 (condition: PPI, PPF) mixed ANOVAs (Figure 6). As expected, the N100 component was significantly reduced in PPI (M ± SE = 0.64 ± 0.23 μ V) compared with PPF (M ± SE= –1.44 ± 0.27 μ V) (main effect of condition: $F\left(1,61\right)=35.78;p<0.001;\eta_{p}^{2}=0.37$). Furthermore, the P200 was significantly enhanced in PPF (M ± SE = 4.25 ± 0.38 μ V) relative to PPI (M ± SE= 2.05 ± 0.28 μ V) (main effect of condition: $F\left(1,61\right)=40.36;p<0.001;\eta_{p}^{2}=0.40$). There was no significant effect of group or interaction between the variables (*p* > 0.05). Finally, there was no significant effect on the P50 component or on the P50, N100, or P200 latencies (all *p*’s > 0.21).

### First-Derivative Potentials

**FIGURE 7:**
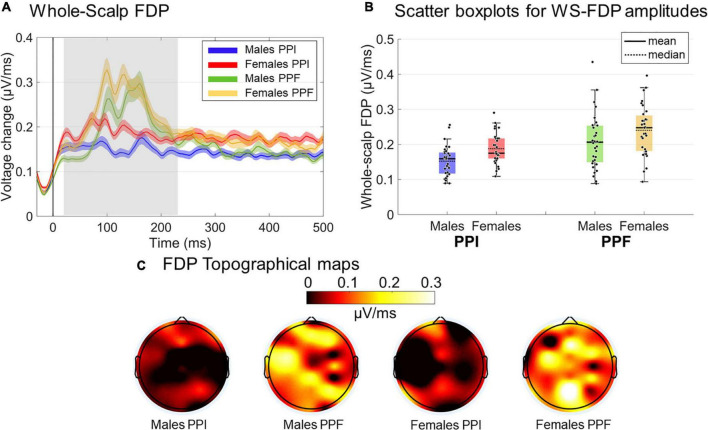
Grand-averaged FDP responses for both groups and conditions. **(A)** The time course of the WS-FDP responses is illustrated for males (PPI: blue, PPF: green) and females (PPI: red, PPF: orange), respectively, from –30 to 500 ms (time 0 is the startle onset). Color-shaded areas correspond to the time course of the standard error (± 1 SE) of the mean. **(B)** The descriptive statistics (mean and median) of groups/conditions are depicted as scatter boxplots. **(C)** Topographical maps for averaged FDP during 20–230 ms poststartle.

WS-FDP measures were also contrasted in the 20- to 240-ms poststartle window (Figure 7). Regarding the WS-FDP amplitudes, PPI (M ± SE = 0.169 ± .005 μ V/ms) was decreased overall relative to PPF (M ± SE = 0.223 ± 0.009 μ V/ms) responses [main effect of condition: $F\left(1,61\right)=46.42;p<0.001;\eta_{p}^{2}=0.432$]. Females (M ± SE= 0.216 ± 0.009 μ V/ms) showed significantly higher WS-FDPs than males (M ± SE = 0.177± 0.009 μ V/ms) (main effect of group: $F\left(1,61\right)=8.72;p=0.004;\eta_{p}^{2}=0.125$), suggesting an overall high degree of rapid fluctuations immediately after startle onset for the former. There was no significant interaction between the variables (*p*0.756). No significant outcomes were yielded for WS-FDP latencies (all *p*’s > 0.28).

### Relationship Between Acoustic Startle Response and Age

**FIGURE 8:**
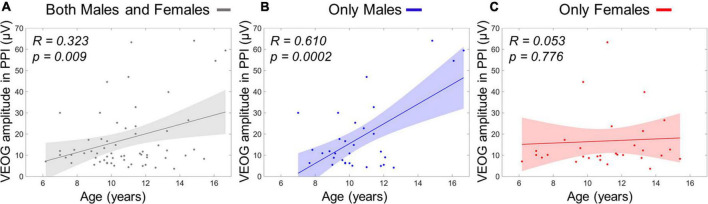
Linear term of VEOG amplitudes in PPI correlating with age as scatterplots. **(A)** Age versus VEOG amplitudes in PPI in the whole population. **(B)** Age versus VEOG amplitudes in PPI in Males only. **(C)** Age versus VEOG amplitudes in PPI in females only. Color-shaded areas indicate the 95% confidence interval of the best-fitting line.

The stepwise regression model for predicting age using four VEOG predictors (*X*_1_: VEOG amplitudes of all subjects in PPI, *X*_2_: VEOG amplitudes of all subjects in PPF, *X*_3_: VEOG latencies of all subjects in PPI, *X*_4_: VEOG latencies of all subjects in PPF) revealed only the *X*_1_ as significant linear predictor of age. The model showed a significant overall fit of age∼ 9.98+0.05×X_1_;R^2^=0.11; p=0.009. To test potential group effects in the age model of the whole population, two different SMLRs were also conducted with the same dependent variable (i.e., age) and predictors, separately for males and females. The model for males maintained the *X*_1_ as significant linear predictor (overall fit: age_males_∼9.14+0.08*times*X_1,males_;R^2^=0.37; p<0.001), whereas that of females did not show any significant predictor. These outcomes suggest that the positive correlation between VEOG responses in PPI and age, which was identified in the whole study population (Figure 8A), is primarily attributed to the males (Figure 8B). Such correlation is not presented in the females, as shown in Figure 8C.

**FIGURE 9:**
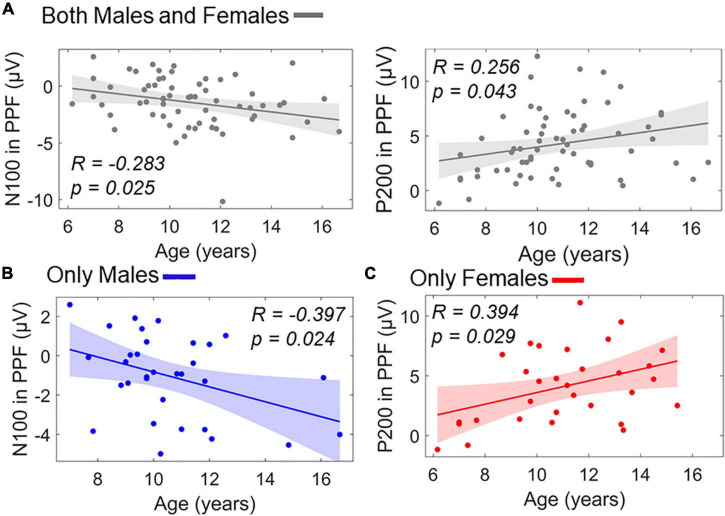
Linear terms of ERP amplitudes in PPF correlating with age as scatterplots. **(A)** Age versus N100 in PPF (left) and age versus P200 in PPF (right) in the whole population. **(B)** Age versus N100 in PPF for males subgroup. **(C)** Age versus P200 in PPF for females subgroup. Color-shaded areas indicate the 95% confidence interval of the best-fitting line.

Similar stepwise regression model for predicting age using the ERP responses as predictors (*X*_1_/*X*_2_: P50 amplitude in PPI/PPF, *X*_3_/*X*_4_: N100 amplitude in PPI/PPF, *X*_5_/*X*_6_: P200 amplitude in PPI/PPF) revealed two significant linear predictors of age, namely, *X*_4_ and *X*_6_. The model showed a significant overall fit of age∼9.53–0.30×X_4_+0.20×X_6_;R^2^=0.14; p=0.009. Figure 9A shows separately the scatterplots for both predictors of age in the whole population. To investigate potential intragender effects, separate validation models were conducted for males and females, revealing *X*_4_ as significant predictor for males’ age (overall fit: age_males_∼10.16–0.42×X_4,males_;R^2^=0.16; p=0.025) and *X*_6_ as significant predictor for females’ age (overall fit: age_females_∼9.73+0.32×X_6,females_;R^2^=0.15; p=0.028) (Figure 9).

There were no significant linear predictive terms for whole-group or intragroup age when considering the WS-FDP measures as predictors (all p’s>0.33).

### Relationship Between the Acoustic Startle Response and Behavioral Scales

Multipredictor models were also conducted to investigate predictive capabilities of the extracted VEOG/EEG measures on behavioral and perceptual skill scales. To reduce the number of the conducted SMLRs and multiple comparisons, VEOG, ERP, and FDP responses were all included as predictors. Model for predicting sex and CBCL and WISC-III subscales were conducted, and the related outcomes are presented in Table 2.

**TABLE 2:** Stepwise multiple linear models for predicting gender and WISC-III and CBCL scores.

Measure	Dependent variable	Significant predictor(s)	β Coefficient(s)		Overall fit	
			β	*p* value	R^2^	*p* value
	Sex	WS-FDP in PPI	4.23	0.002	0.151	0.002
CBCL	Attention problems	n.s.	—	—	—	—
	Internalizing	N100 in PPI	–1.35	0.024	0.080	0.024
	Externalizing	n.s.	—	—	—	—
WISC-III	Picture completion	N100 in PPF	–0.38	0.018	0.090	0.018
	Similarities	n.s.	—	—	—	—
	Arithmetic	n.s.	—	—	—	—
	Block design	N100 in PPF	–0.31	0.048	0.070	0.048
	Full-scale IQ	n.s.	—	—	—	—

*n.s., non-significant predictors.*

As expected from the identified main effect of group in WS-FDP, sex was linearly associated with WS-FDP. Predictor significance was yielded only for PPI and not PPF WS-FDPs. In CBCL scales, there was a positive correlation (i.e., more negative N100 values) of internalizing scores and N100 amplitudes in PPI, whereas attention problems and externalizing behavior did not show any significant predictors. To investigate the impact of age in predicting the internalizing scores, we reran an identical SMLR model by additionally considering the age as predictor. Again, only the N100 in PPI was the significant linear predictor of internalizing (β coefficient = –1.35; *p* = 0.024; *R*^2^ = 0.080).

Arithmetic, similarities, and full-scale IQ tests of WISC-III were not related with VEOG/EEG amplitude strength, whereas the nonverbal perceptual skills (picture completion and block design) showed positive associations with N100 amplitude in PPI. Note that block design demonstrated marginal significance (*p* = 0.048), while also picture completion and block design scores were intercorrelated (*r* = 0.6).

## Discussion

To review, this study investigated electrophysiological (ocular and EEG) responses in a PPI and PPF paradigm in children and adolescents. To the best of the authors’ knowledge, our study is the first to examine associations between PPI/PPF measures and sex, age, as well as behavior characteristics in a wide range of ages from 6 to 17 years. Across all electrophysiological measures, neural inhibition was observed in the PPI but not in the PPF condition, extending existing knowledge about the block-out of sensory processing in the ASR to healthy young populations. Further, analysis of WS-FDPs provided evidence for sex differences in inhibitory mechanisms, whereas more location-precise (such as ERPs and VEOG) measures were not sensitive to sex effects. We also identified positive correlations between electromyographic (EMG) amplitudes in PPI and N100/P200 amplitudes in PPF, indicating increasingly maturated ASR in youth ages. The EMG aging effect was pronounced in males, whereas females’ EMG responses did not show age correlations. Given the group differences, WS-FDP in PPI exhibited linear correlations with sex, with females presenting enhanced PPI (and thus weaker inhibition) than males. Attention-related N100 component was also associated with enhanced internalizing (of CBCL) and nonverbal perceptual skills (picture completion and block design of WISC-III), whereas P50 and P200 did not reveal any behavioral association with participants’ screening measures.

The common ground truth about PPI and PPF mechanisms holds that PPF refers to enhancements of ASR, whereas PPI is associated with diminishment of startle reaction. The presented eyeblink enhancements in PPF are in line with extensive research on EMG-oriented ASRs. This is mainly attributed to the inherent mechanisms underlying PPI, where the sensorimotor gating is responsible for preventing sensory overload. The enabling factor for inhibiting an upcoming stimulus (startle) relies on the ongoing processing of an initial prepulse, leading to a partial sensory avoidance of the disruptive pulse (Geyer et al., 1990; Wynn et al., 2005). On the contrary, PPF is linked to sustained attention and orienting attention (Dawson et al., 1997). To support these statements, it has been observed that cardiac rates slow down when prepulse–pulse interval is long (as in PPF), presumably indicating awareness and attention guidance to the upcoming startle (Graham, 1975; Eder et al., 2009). As a result, our findings regarding reduced VEOG responses in PPI confirm identical conclusions reported in adult populations (Wynn et al., 2005; Kofler et al., 2013; Aasen et al., 2016) and suggest reduced muscular excitability due to the short prepulse–pulse pairing and sensory block-out.

Neural ASRs appeared also in accordance with the “protection-of-processing” theory (Graham, 1975), which justifies the PPI/PPF function: the prepulse–pulse stimulation enables simultaneous cognitive processing for both the weak prepulse and the gating mechanism that dampens the information of the intense disruptive input. Indeed, the early ASR-elicited ERP components (N100 and P200) were observed reduced in PPI prestimulation, proposing a temporal-specific attentional prevention of sensory overload. In general, N100 has been linked with early selective attention processes, showing reduced amplitudes to the nonattended tasks compared with the attended ones (Näätänen and Picton, 1987; Fujiwara et al., 1998). Early negativities approximately 100 ms have been also proposed as indices of attention orienting to the auditory stimuli in passive listening tasks, improving the discrimination between signal and noise (Näätänen and Picton, 1987), thereby contributing into efficient cognitive functioning. A lack of N100 increase during allocation of attention provides also corroborating evidence for reduced N100 associated with impaired attention processes (Rosburg et al., 2008). Similarly, P200 component has also been coupled with N100 role in attention allocation, reflecting task-relevant stimulus salience (Yuan et al., 2009). Thus, the combination of two ERP decrements during PPI can be explained as attentional denoising of task-irrelevant information, filtering out the startle processing to protect the ongoing attended processing on the prepulse.

PPI-elicited neural substrates have explained inhibitory sexual dimorphism in both healthy and clinical groups and have been proven as a key factor that discriminates males and females (Ellwanger et al., 2003; Kumari et al., 2004; Naysmith et al., 2021). Here we found that ROI-specific ERP could not discriminate between genders, but the WS poststartle FDPs could indicate more abrupt excitability in females than males. Common findings report weaker sensorimotor gating in women than men, when the EMG- or neural-oriented inhibition index is computed as difference between the prepulse–pulse ASR and pulse-alone ASR trials. This is compatible with the presented enhanced PPI ASRs (i.e., less block-out of the startle tone) in females (Kumari et al., 2004; Aasen et al., 2016; Abel et al., 2016). The dominant substance that can account for gender differences is the different estrogen production levels among genders, which are engaged in sensorimotor gating operation (Koch, 1998; Becker, 2000). It is believed that estrogen influences neurochemical dynamics, especially dopaminergic activity (Becker, 2000), in the nucleus accumbens involved in gating and overload prevention (Aasen et al., 2016). Hormonal instabilities have been also noticed as a crucial factor in females’ PPI diminishments, influencing also cognitive performance (Maki et al., 2002) and females’ ASR test–retest reproducibility (Jovanovic et al., 2004; Stachtea et al., 2020). Our findings support the hypothesis of sexual dimorphism in inhibitory function, even from early life stages (<18 years), reflected by the global, baseline-robust FDPs, whereas ERPs failed to show biological sex alterations, as in (Freedman et al., 1987; Waldo et al., 1987).

Given that age influences a variety of behavioral and psychophysiological markers of cognitive processing, it comprises a potential confounding source for the startle response (Naysmith et al., 2021). Contradictory to early research that suggested a global decline in inhibitory function with normal aging (Cerella, 1985), a study by Ellwanger et al. (2003) demonstrated an inverted U-shaped function with age (greatest PPI at intermediate ages) in a normal population aged 18–88 years. In a study with children, increasing levels of ASR maturation in the range of 6–18 years was found. Our findings show a positive correlation between age and VEOG amplitude in the male participants only, potentially due to the limited ASR reliability in females due to increased intragroup fluctuations in ASR with age (Stachtea et al., 2020). Considering our low sample size per year of age as well as the restricted age range of our sample, we believe that further studies need to be conducted in order to investigate age maturation of the ASR modulation, using a population with a wider age range, from childhood to senility.

With regard to the neural measures, we found that the N100 and the P200 components were also correlated with age. More specifically, in the PPF condition, N100 amplitude was negatively correlated with age in males, whereas P200 was positively correlated with age in females. Considering that PPF is associated with facilitated processing of incoming stimuli, the direction of the effects might suggest that PPF improves with age. This is in line with previous studies showing that maturation of PPI of the startle reflex increases continuously from 3 to 10 years old, reaching adult levels at approximately 9–10 years old (Gebhardt et al., 2012). Previous studies demonstrated full maturity of the ASR at 8 years old; however, no older children were investigated (Ornitz et al., 1986, 1991). Considering that the aforementioned studies investigated VEOG responses separately children versus adults, our study is the first to examine and demonstrate maturation effects of the startle responses at the neural level from childhood to adolescence. Interestingly, ASR seems to maturate throughout these ages, without reaching a plateau. Future studies need to investigate neural ASR maturation extending from childhood throughout adulthood.

Our regression analysis revealed that the N100 amplitude in PPI was correlated with internalizing scores at the CBCL. Specifically, we observed that children and adolescents high in internalizing exhibited stronger N100 (more negative) to the PPI condition compared with individuals with low internalizing scores. Notably, it is difficult to compare our findings to previous research, as ASR responses have not been previously associated with internalizing. However, our findings are consistent with literature demonstrating differentiated brain patterns depending on the level of internalizing (Stewart et al., 2014; Auerbach et al., 2015; Macnamara et al., 2016; Speed et al., 2016; Bunford et al., 2017; Feldmann et al., 2018). For example, frontal alpha asymmetry, that is, difference between EEG alpha power in the frontal left versus right hemisphere, is lower in high internalizing individuals, potentially associated with more withdrawal or less approach motivation characteristics (Stewart et al., 2014; Feldmann et al., 2018). Furthermore, anxiety and depressive symptoms are associated with enhanced late positive potential evoked responses to emotional stimuli in adolescence and adulthood (Macnamara et al., 2016; Bunford et al., 2017; McLean et al., 2020), especially in response to negatively valenced self-referential words (Auerbach et al., 2015; Speed et al., 2016).

Altogether, our findings seem to be related to the negative potentiation hypothesis, which poses that negative mood induces enhanced responses to negatively valenced cues in the environment (Beck, 1976). In particular, the ASR constitutes an involuntary response triggered by a sudden “threatening” stimulus. In the PPI condition, the ASR to the second tone is normally suppressed. However, in line with the negative potentiation hypothesis, internalizing problems might be associated with more alertness, that is, decreased suppression of threatening stimuli. In our study, this was potentially reflected in enhanced N100 responses in the PPI condition for high internalizing individuals. This finding is also in line with the previously reported attention bias for negative stimuli in depressed children and adolescents (see (Platt et al., 2016) for a review). Another explanation could be that more negative N100 amplitude in the PPI condition for high internalizing children and adolescents might be due to an increased sensitivity to external sources of threat for anxious/depressed people (Tobias and Ito, 2021). Reduced PPI (i.e., more negative N100) may therefore increase responsiveness to novel, startling stimuli enhancing receptivity to sudden input.

Finally, our effect was identified in an early time window, suggesting that this process of lower ASR during suppression of a startle tone in high internalizing individuals might be automatic. It is worth noting that further studies need to investigate whether this N100 response develops in conjunction with other dysregulations in neural system. Last but not least, it is important to note that by testing a sample of healthy individuals, our study is free from the confounding factor of clinical cases, where it is hard to decipher whether the effects are due to comorbidity.

With regard to intelligence, performance on picture completion and block design subscales was found to be negatively correlated with N100 amplitude in PPF, that is, stronger N100 component. Interestingly, both subscales tap onto practical, nonverbal abilities, namely, speed of object recognition and spatial visuomotor skills, respectively. According to previous studies, the Wechsler intelligence scores are usually positively correlated with neurophysiological efficiency (Robaey et al., 1995; Deary and Caryl, 1997). More specifically, PPI increases with maturation from childhood to adolescence (Ornitz et al., 1986). The reason is that PPI may help maintain the focus of attention to a sensory stimulus and not get interrupted by a close-following process (Norris and Blumenthal, 1996), leading to more efficient sensory processing. In line with this proposition, an enhanced PPF might reflect that sensory processing is more facilitated, which equates to higher neural efficiency. Therefore, children and adolescents with higher nonverbal intelligence might exhibit higher neural efficiency than individuals with lower intelligence, as the same stimulus elicits a stronger facilitatory response.

An alternative explanation could be related to attention differences between individuals with high versus low WISC scores. For instance, children with ADHD have problems with attention and initial stages of memory, as identified using the WISC scale (Mayes et al., 1998). Furthermore, PPF was found to be greater during attended compared with ignored stimuli (Hawk et al., 2002). Therefore, it might be that in our study higher performance at the WISC practical subscales relates to increased attention allocation during the task, thus leading to stronger PPF responses.

This study is not without limitations that can be addressed in the future to elucidate some confounding aspects. Firstly, PPI and PPF were directly contrasted with the absence of a pulse-alone condition. Transforming the PPI and PPF indices using the pulse-alone ASR as baseline could reproduce the presented outcomes, even when intersubject variability has been restricted based on subject-specific pulse-alone reactivity. Furthermore, we acknowledge that the presence of hormonal status data for the female participants could help us better understand whether the hormonal status is a crucial factor of the inhibitory function, even from the early years of life. Another limitation in gender differences is the usage of the WS-FDPs, which exhibit low spatial precision on ASR changes. Because of the inherent low spatial resolution of EEG, another more location-precise technique (e.g., fMRI) could contrast the FDPs of voxels’ activation, especially focused on the corticostriatopallidopontine circuitry that modulates PPI (Swerdlow and Geyer, 1998). The study population may also be examined for participants (especially < 8 years old) showing non-maturated ASRs (Gebhardt et al., 2012), although here we preferred to not exclude such participants for purposes of maintaining adequate statistical power. Repetition of the same experiment in another same-age group is needed also to stabilize the conclusions, given the sensitivity of the ASR-related trials to noise. Finally, although most studies failed to confirm correlations between neural and muscular PPI responses (Kedzior et al., 2006), it is essential to investigate whether neural effects are associated with muscular responses (or *vice versa*). Toward this analysis, it should be noted that an antisymmetric approach is required for simultaneous consideration of neural and muscular analyses (epochs with strong eyeblinks reinforce muscular PPI, but, contradictorily, are removed from neural PPI), thus reducing the power of parallel comparison of these measures. This is mainly the reason of not performing correlations between EMG and EEG responses, suggesting that EMG and EEG ASRs have not calculated over the exact same trials (EEGs are ICA-corrected). It is also to be noted that additional research is needed to reproduce the present VEOG-based findings with surface or needle EMG recording electrodes. Another limitation of the study is that pubertal maturation, through Tanner staging, was not assessed in our participants. In a bigger sample, biomarkers of pre-pubertal males and females might show differences from those of pubertal males and females, respectively, due to the effects of sex-steroid hormones during this period of rapid maturation. Lastly, internalizing symptoms in the CBCL are parent-reported, and self-reported measures, especially in older children, could be more accurate in rating these symptoms.

Overall, clinical research may considerably benefit from the usage of PPI and PPF as biomarkers, whereas a more detailed investigation of their sexual and aging effects in childhood could indicate early development of disorders with PPI and PPF impairments. Mapping the motor/neural ASRs and behavioral protocols in young participants could also allow for developments of a prediagnostic blueprint for normal cognitive/behavioral functioning. To conclude, future research is needed to build a standardized baseline procedure for early identification of neural abnormalities underlying children’s cognition and behavior.

## Data Availability Statement

The raw data supporting the conclusions of this article will be made available by the authors, without undue reservation.

## Ethics Statement

The studies involving human participants were reviewed and approved by the Scientific and Ethics Committee of “Aghia Sophia” and “Aiginiteion” Hospitals. Written informed consent to participate in this study was provided by the participants’ legal guardian/next of kin.

## Author Contributions

AG: formal analysis, writing–original draft, visualization, and software. IZ: investigation, formal analysis, and writing–original draft. PPa: review and editing. PPe: data curation, validation, review, and editing. GM: review and editing. GC: conceptualization and investigation. XS: data curation. CC: validation and supervision. CP: conceptualization, methodology, validation, and investigation. All authors contributed to the article and approved the submitted version.

## Conflict of Interest

The authors declare that the research was conducted in the absence of any commercial or financial relationships that could be construed as a potential conflict of interest.

## Publisher’s Note

All claims expressed in this article are solely those of the authors and do not necessarily represent those of their affiliated organizations, or those of the publisher, the editors and the reviewers. Any product that may be evaluated in this article, or claim that may be made by its manufacturer, is not guaranteed or endorsed by the publisher.

## References

[B1] Aasen I. Kolli L. Kumari V. (2016). Sex effects in prepulse inhibition and facilitation of the acoustic startle response: implications for pharmacological and treatment studies. *J. Psychopharmacol.* 19 39–45. 10.1177/0269881105048890 15671127

[B2] Abel K. Waikar M. Pedro B. Hemsley D. Geyer M. (2016). Repeated testing of prepulse inhibition and habituation of the startle reflex: a study in healthy human controls. *J. Psychopharmacol.* 12 330–337. 10.1177/026988119801200402 10065906

[B3] Achenbach T. (1991). *Manual for the Child Behavior Checklist/4-18 and 1991 Profile.* Burlington, VT: University of Vermont, Department of Psychiatry.

[B4] Achenbach T. Edelbrock C. (1991). *Child Behavior Checklist.* Burlington, VT: Research Center for Children, Youth and Families, 371–392.

[B5] Achenbach T. McConaughy S. Ivanova M. Rescorla L. (2011). *Manual for the ASEBA Brief Problem Monitor (BPM).* Burlington, VT: ASEBA, 33.

[B6] Auerbach R. P. Stanton C. H. Proudfit G. H. Pizzagalli D. A. (2015). Self-referential processing in depressed adolescents: a high-density ERP study. *J. Abnorm. Psychol.* 124:233. 10.1037/abn0000023 25643205 PMC4429006

[B7] Balaban M. T. Anthony B. J. Graham F. K. (1989). Prestimulation effects on blink and cardiac reflexes of 15-month human infants. *Dev. Psychobiol.* 22 115–127. 10.1002/dev.420220203 2925000

[B8] Barlow D. H. (2000). Unraveling the mysteries of anxiety and its disorders from the perspective of emotion theory. *Am. Psychol.* 55 1247–1263. 10.1037//0003-066x.55.11.1247 11280938

[B9] Beck A. (1976). *Cognitive Therapy and the Emotional Disorders.* New York, NY: International Universities Press, 356.

[B10] Becker J. (2000). Oestrogen effects on dopaminergic function in striatum. *Neuronal Cogn. Eff. oestrogens* 230 134–145. 10.1002/0470870818.ch10 10965506

[B11] Blumenthal T. D. Cuthbert B. N. Filion D. L. Hackley S. Lipp O. V. Van Boxtel A. (2005). Committee report: guidelines for human startle eyeblink electromyographic studies. *Psychophysiology* 42 1–15. 10.1111/j.1469-8986.2005.00271.x 15720576

[B12] Braff D. L. Geyer M. A. Swerdlow N. R. (2001). Human studies of prepulse inhibition of startle: normal subjects, patient groups, and pharmacological studies. *Psychopharmacology (Berl)* 156 234–258. 10.1007/s002130100810 11549226

[B13] Bunford N. Kujawa A. Fitzgerald K. D. Swain J. E. Hanna G. L. Koschmann E. (2017). Neural reactivity to angry faces predicts treatment response in pediatric anxiety. *J. Abnorm. Child Psychol.* 45:385. 10.1007/s10802-016-0168-2 27255517 PMC5800984

[B14] Buse J. Beste C. Herrmann E. Roessner V. (2015). Neural correlates of altered sensorimotor gating in boys with Tourette Syndrome: a combined EMG/fMRI study. *World J. Biol. Psychiatry* 17 187–197. 10.3109/15622975.2015.1112033 26624257

[B15] Cerella J. (1985). Information processing rates in the elderly. *Psychol. Bull.* 98 67–83. 10.1037/0033-2909.98.1.67 4034819

[B16] Chaumon M. Bishop D. V. M. Busch N. A. (2015). A practical guide to the selection of independent components of the electroencephalogram for artifact correction. *J. Neurosci. Methods* 250 47–63. 10.1016/j.jneumeth.2015.02.025 25791012

[B17] Davis M. Gendelman D. Tischler M. Gendelman P. (1982). A primary acoustic startle circuit: lesion and stimulation studies. *J. Neurosci.* 2 791–805. 10.1523/JNEUROSCI.02-06-00791.1982 7086484 PMC6564345

[B18] Davis M. Hitchcock J. M. Rosen J. B. (1988). Anxiety and the amygdala: pharmacological and anatomical analysis of the fear-potentiated startle paradigm. *Psychol. Learn. Motiv. Adv. Res. Theory* 21 263–305. 10.1016/s0079-7421(08)60031-6

[B19] Dawson M. Schell A. Swerdlow N. (1997). “Cognitive, clinical, and neurophysiological implications of startle modification,” in *Attention and orienting: Sensory and motivational processes*, eds Lang P. J. Simons R. F. Balaban M. T. (Mahwah, NJ: Lawrence Erlbaum Associates Publishers), 257–279.

[B20] Dawson M. E. Hazlett E. A. Filion D. L. Nuechterlein K. H. Schell A. M. (1993). Attention and schizophrenia: impaired modulation of the startle reflex. *J. Abnorm. Psychol.* 102 633–641. 10.1037//0021-843x.102.4.633 8282934

[B21] De Pascalis V. Cozzuto G. Russo E. (2013). Effects of personality trait emotionality on acoustic startle response and prepulse inhibition including N100 and P200 event-related potential. *Clin. Neurophysiol.* 124 292–305. 10.1016/j.clinph.2012.07.018 22938794

[B22] Deary I. J. Caryl P. G. (1997). Neuroscience and human intelligence differences. *Trends Neurosci.* 20 365–371. 10.1016/s0166-2236(97)01070-9 9246731

[B23] Delorme A. Makeig S. (2004). EEGLAB: an open source toolbox for analysis of single-trial EEG dynamics including independent component analysis. *J. Neurosci. Methods* 134 9–21. 10.1016/j.jneumeth.2003.10.009 15102499

[B24] Eder D. N. Elam M. Wallin B. G. (2009). Sympathetic nerve and cardiovascular responses to auditory startle and prepulse inhibition. *Int. J. Psychophysiol.* 71 149–155. 10.1016/j.ijpsycho.2008.09.001 18824200

[B25] Ellwanger J. Geyer M. A. Braff D. L. (2003). The relationship of age to prepulse inhibition and habituation of the acoustic startle response. *Biol. Psychol.* 62 175–195. 10.1016/s0301-0511(02)00126-6 12633977

[B26] Feldmann L. Piechaczek C. E. Grünewald B. D. Pehl V. Bartling J. Frey M. (2018). Resting frontal EEG asymmetry in adolescents with major depression: Impact of disease state and comorbid anxiety disorder. *Clin. Neurophysiol.* 129 2577–2585. 10.1016/j.clinph.2018.09.028 30415151

[B27] Freedman R. Adler L. E. Waldo M. (1987). Gating of the auditory evoked potential in children and adults. *Psychophysiology* 24 223–227. 10.1111/j.1469-8986.1987.tb00282.x 3602274

[B28] Fujiwara N. Nagamine T. Imai M. Tanaka T. Shibasaki H. (1998). Role of the primary auditory cortex in auditory selective attention studied by whole-head neuromagnetometer. *Cogn. Brain Res.* 7 99–109. 10.1016/s0926-6410(98)00014-7 9774711

[B29] Gebhardt J. Schulz-Juergensen S. Eggert P. (2012). Maturation of prepulse inhibition (PPI) in childhood. *Psychophysiology* 49 484–488. 10.1111/j.1469-8986.2011.01323.x 22176532

[B30] Georgas D. Paraskevopoulos I. Bezevegis I. Giannitsas N. (1997). *Greek WISC-III: Wechsler Intelligence Scales for Children.* Athens: Ellinika Grammata.

[B31] Geyer M. A. Swerdlow N. R. Mansbach R. S. Braff D. L. (1990). Startle response models of sensorimotor gating and habituation deficits in schizophrenia. *Brain Res. Bull.* 25 485–498. 10.1016/0361-9230(90)90241-q 2292046

[B32] Giannopoulos A. E. Zioga I. Papageorgiou P. C. Kapsali F. Spantideas S. T. Kapsalis N. C. (2021b). Early auditory-evoked potentials in body dysmorphic disorder: an ERP/sLORETA study. *Psychiatry Res.* 299:113865. 10.1016/j.psychres.2021.113865 33735739

[B33] Giannopoulos A. Spantideas S. Capsalis C. Papageorgiou P. Kapsalis N. Kontoangelos K. (2021a). Instantaneous radiated power of brain activity: application to prepulse inhibition and facilitation for body dysmorphic disorder. *Biomed. Eng. Online* 20:108. 10.1186/s12938-021-00946-9 34689781 PMC8543766

[B34] Graham F. Strock B. Zeigler B. (2015). “Excitory and inhibitory influences on reflex responsiveness,” in *Aspects of the Development of Competence*, ed. Andrew Collins W. (Hillsdale, NJ: L. Erlbaum Associates), 13–49.

[B35] Graham F. K. (1975). The more or less startling effects of weak prestimulation. *Psychophysiology* 12 238–248. 10.1111/j.1469-8986.1975.tb01284.x 1153628

[B36] Grillon C. (2007). Models and mechanisms of anxiety: evidence from startle studies. *Psychopharmacology (Berl)* 199 421–437. 10.1007/s00213-007-1019-1 18058089 PMC2711770

[B37] Hawk L. W. Pelham W. E. Yartz A. R. (2002). Attentional modification of short-lead prepulse inhibition and long-lead prepulse facilitation of acoustic startle among preadolescent boys. *Psychophysiology* 39 333–339. 10.1017/s0048577201393071 12212652

[B38] Henderson D. A. Denison D. R. (1989). Stepwise regression in social and psychological research. *Psychol. Rep.* 64 251–257. 10.2466/pr0.1989.64.1.251

[B39] Hetrick W. P. Sandman C. A. Bunney W. E. Jin Y. Potkin S. G. White M. H. (1996). Gender differences in gating of the auditory evoked potential in normal subjects. *Biol. Psychiatry* 39 51–58. 10.1016/0006-3223(95)00067-4 8719126

[B40] Jafari Z. Kolb B. E. Mohajerani M. H. (2020). Prepulse inhibition of the acoustic startle reflex and P50 gating in aging and Alzheimer’s disease. *Ageing Res. Rev.* 59:101028. 10.1016/j.arr.2020.101028 32092463

[B41] Jovanovic T. Szilagyi S. Chakravorty S. Fiallos A. M. Lewison B. J. Parwani A. (2004). Menstrual cycle phase effects on prepulse inhibition of acoustic startle. *Psychophysiology* 41 401–406. 10.1111/1469-8986.2004.00166.x 15102125

[B42] Kappenman E. S. Luck S. J. (2016). Best practices for event-related potential research in clinical populations. *Biol. Psychiatry Cogn. Neurosci. Neuroimaging* 1 110–115. 10.1016/j.bpsc.2015.11.007 27004261 PMC4797328

[B43] Kaufman A. S. Kaufman J. C. Balgopal R. McLean J. E. (1996). Comparison of three WISC-III short forms: weighing psychometric, clinical, and practical factors. *J. Clin. Child Psychol.* 25 97–105. 10.1207/s15374424jccp2501_11

[B44] Kedzior K. K. Koch M. Basar-Eroglu C. (2006). Prepulse inhibition (PPI) of auditory startle reflex is associated with PPI of auditory-evoked theta oscillations in healthy humans. *Neurosci. Lett.* 400 246–251. 10.1016/j.neulet.2006.02.048 16530955

[B45] Koch M. (1998). Sensorimotor gating changes across the estrous cycle in female rats. *Physiol. Behav.* 64 625–628. 10.1016/s0031-9384(98)00098-5 9817573

[B46] Kofler M. Kumru H. Schaller J. Saltuari L. (2013). Blink reflex prepulse inhibition and excitability recovery: influence of age and sex. *Clin. Neurophysiol.* 124 126–135. 10.1016/j.clinph.2012.07.001 22857876

[B47] Kponee-Shovein K. Z. Grashow R. Coull B. A. Téllez-Rojo M. M. Schnaas L. del Carmen Hernández-Chávez M. (2019). Socio-demographic predictors of prepulse inhibition: a prospective study in children and adolescents from Mexico City. *Biol. Psychol.* 145 8–16. 10.1016/j.biopsycho.2019.03.003 30940478 PMC12042598

[B48] Kponee-Shovein K. Z. Weisskopf M. G. Grashow R. Rotem R. S. Coull B. A. Schnaas L. (2020). Estimating the causal effect of prenatal lead exposure on prepulse inhibition deficits in children and adolescents. *Neurotoxicology* 78 116–126. 10.1016/j.neuro.2020.02.013 32126243 PMC12042602

[B49] Krol N. P. C. M. De Bruyn E. E. J. Coolen J. C. van Aarle E. J. M. (2006). From CBCL to DSM: a comparison of two methods to screen for DSM-IV diagnoses using CBCL data. *J. Clin. Child Adolesc. Psychol.* 35 127–135. 10.1207/s15374424jccp3501_11 16390308

[B50] Kumari V. Aasen I. Sharma T. (2004). Sex differences in prepulse inhibition deficits in chronic schizophrenia. *Schizophr. Res.* 69 219–235. 10.1016/j.schres.2003.09.010 15469195

[B51] Kumari V. Antonova E. Zachariah E. Galea A. Aasen I. Ettinger U. (2005). Structural brain correlates of prepulse inhibition of the acoustic startle response in healthy humans. *Neuroimage* 26 1052–1058. 10.1016/j.neuroimage.2005.03.002 15961045

[B52] Kumari V. Gray J. A. Gupta P. Luscher S. Sharma T. (2003). Sex differences in prepulse inhibition of the acoustic startle response. *Pers. Individ. Dif.* 35 733–742. 10.1016/s0191-8869(02)00266-0

[B53] Kumari V. Konstantinou J. Papadopoulos A. Aasen I. Poon L. Halari R. (2010). Evidence for a role of progesterone in menstrual cycle-related variability in prepulse inhibition in healthy young women. *Neuropsychopharmacology* 35 929–937. 10.1038/npp.2009.195 19956084 PMC3055354

[B54] Li L. Du Y. Li N. Wu X. Wu Y. (2009). Top–down modulation of prepulse inhibition of the startle reflex in humans and rats. *Neurosci. Biobehav. Rev.* 33 1157–1167. 10.1016/j.neubiorev.2009.02.001 19747594

[B55] Liu Y. Lin J. (2019). A general-purpose signal processing algorithm for biological profiles using only first-order derivative information. *BMC Bioinformatics* 20:611. 10.1186/s12859-019-3188-4 31775621 PMC6882060

[B56] Luck S. J. Gaspelin N. (2017). How to get statistically significant effects in any ERP experiment (and why you shouldn’t). *Psychophysiology* 54 146–157. 10.1111/psyp.12639 28000253 PMC5178877

[B57] Macnamara A. Kotov R. Hajcak G. (2016). Diagnostic and symptom-based predictors of emotional processing in generalized anxiety disorder and major depressive disorder: an event-related potential study. *Cognit. Ther. Res.* 40 275–289. 10.1007/s10608-015-9717-1 27346901 PMC4916772

[B58] Majumdar K. (2012). Differential operator in seizure detection. *Comput. Biol. Med.* 42 70–74. 10.1016/j.compbiomed.2011.10.010 22104594

[B59] Maki P. M. Rich J. B. Shayna Rosenbaum R. (2002). Implicit memory varies across the menstrual cycle: estrogen effects in young women. *Neuropsychologia* 40 518–529. 10.1016/s0028-3932(01)00126-9 11749982

[B60] Mayes S. D. Calhoun S. L. Crowell E. W. (1998). WISC-III freedom from distractibility as a measure of attention in children with and without attention deficit hyperactivity disorder. *J. Atten. Disord.* 2 217–227. 10.1177/108705479800200402

[B61] McLean M. A. Van den Bergh B. R. H. Baart M. Vroomen J. van den Heuvel M. I. (2020). The late positive potential (LPP): a neural marker of internalizing problems in early childhood. *Int. J. Psychophysiol.* 155 78–86. 10.1016/j.ijpsycho.2020.06.005 32561354

[B62] McNaughton N. Corr P. J. (2004). A two-dimensional neuropsychology of defense: fear/anxiety and defensive distance. *Neurosci. Biobehav. Rev.* 28 285–305. 10.1016/j.neubiorev.2004.03.005 15225972

[B63] Mognon A. Jovicich J. Bruzzone L. Buiatti M. (2011). ADJUST: an automatic EEG artifact detector based on the joint use of spatial and temporal features. *Psychophysiology* 48 229–240. 10.1111/j.1469-8986.2010.01061.x 20636297

[B64] Motti-Stefanidi F. Tsiantis J. Richardson S. C. (1993). Epidemiology of behavioural and emotional problems of primary schoolchildren in Greece. *Eur. Child Adolesc. Psychiatry* 2 111–118. 10.1007/BF02098866 29871454

[B65] Näätänen R. Picton T. (1987). The N1 wave of the human electric and magnetic response to sound: a review and an analysis of the component structure. *Psychophysiology* 24 375–425. 10.1111/j.1469-8986.1987.tb00311.x 3615753

[B66] Naysmith L. F. Kumari V. Williams S. C. R. (2021). Neural mapping of prepulse-induced startle reflex modulation as indices of sensory information processing in healthy and clinical populations: a systematic review. *Hum. Brain Mapp.* 42 5495–5518. 10.1002/hbm.25631 34414633 PMC8519869

[B67] Nolan H. Whelan R. Reilly R. B. (2010). FASTER: fully automated statistical thresholding for EEG artifact rejection. *J. Neurosci. Methods* 192 152–162. 10.1016/j.jneumeth.2010.07.015 20654646

[B68] Norris C. M. Blumenthal T. D. (1996). A relationship between inhibition of the acoustic startle response and the protection of prepulse processing. *Psychobiology* 24 160–168. 10.3758/bf03331968

[B69] Ornitz E. M. Guthrie D. Kaplan A. R. Lane S. J. Norman R. J. (1986). Maturation of startle modulation. *Psychophysiology* 23 624–634. 10.1111/j.1469-8986.1986.tb00681.x 3823337

[B70] Ornitz E. M. Guthrie D. Sadeghpour M. Sugiyama T. (1991). Maturation of prestimulation-induced startle modulation in girls. *Psychophysiology* 28 11–20. 10.1111/j.1469-8986.1991.tb03381.x 1886959

[B71] Ornitz E. M. Hanna G. L. De Traversay J. (1992). Prestimulation-induced startle modulation in attention-deficit hyperactivity disorder and nocturnal enuresis. *Psychophysiology* 29 437–451. 10.1111/j.1469-8986.1992.tb01717.x 1410175

[B72] Patterson J. V. Hetrick W. P. Boutros N. N. Jin Y. Sandman C. Stern H. (2008). P50 sensory gating ratios in schizophrenics and controls: a review and data analysis. *Psychiatry Res.* 158 226–247. 10.1016/j.psychres.2007.02.009 18187207

[B73] Platt B. Waters A. M. Schulte-Koerne G. Engelmann L. Salemink E. (2016). A review of cognitive biases in youth depression: attention, interpretation and memory. *Cogn. Emot.* 31 462–483. 10.1080/02699931.2015.1127215 26785312

[B74] Regier D. A. Kuhl E. A. Kupfer D. J. (2013). The DSM-5: classification and criteria changes. *World Psychiatry* 12 92–98. 10.1002/wps.20050 23737408 PMC3683251

[B75] Robaey P. Cansino S. Dugas M. Renault B. (1995). A comparative study of ERP correlates of psychometric and piagetian intelligence measures in normal and hyperactive children. *Electroencephalogr. Clin. Neurophysiol.* 96 56–75. 10.1016/0013-4694(94)00174-j 7530189

[B76] Rosburg T. Boutros N. N. Ford J. M. (2008). Reduced auditory evoked potential component N100 in schizophrenia — a critical review. *Psychiatry Res.* 161 259–274. 10.1016/j.psychres.2008.03.017 18926573

[B77] Roussos A. Karantanos G. Richardson C. Hartman C. Karajiannis D. Kyprianos S. (1999). Achenbach’s child behavior checklist and teachers’ report form in a normative sample of Greek children 6–12 years old. *Eur. Child Adolesc. Psychiatry* 8 165–172. 10.1007/s007870050125 10550697

[B78] San-Martin R. Zimiani M. I. Noya C. Ávila M. A. V. Shuhama R. Del-Ben C. M. (2018). A method for simultaneous evaluation of muscular and neural prepulse inhibition. *Front. Neurosci.* 12:654. 10.3389/fnins.2018.00654 30319337 PMC6168667

[B79] Siddle D. A. T. (1991). Orienting, habituation, and resource allocation: an associative analysis. *Psychophysiology* 28 245–259. 10.1111/j.1469-8986.1991.tb02190.x 1946891

[B80] Speed B. C. Nelson B. D. Auerbach R. P. Klein D. N. Hajcak G. (2016). Depression risk and electrocortical reactivity during self-referential emotional processing in 8 to 14 year-old girls. *J. Abnorm. Psychol.* 125:607. 10.1037/abn0000173 27175985 PMC4925302

[B81] Stachtea X. Zioga I. Giannopoulos A. E. Papageorgiou P. C. Spantideas S. T. Kapsalis N. C. (2020). Test-retest reliability of brain oscillations in a prepulse inhibition and facilitation paradigm: effects of gender in healthy humans. *Neuroreport* 31 985–990. 10.1097/WNR.0000000000001503 32694313

[B82] Stewart J. L. Coan J. A. Towers D. N. Allen J. J. B. (2014). Resting and task-elicited prefrontal EEG alpha asymmetry in depression: support for the capability model. *Psychophysiology* 51 446–455. 10.1111/psyp.12191 24611480 PMC3984363

[B83] Swerdlow N. Braff D. Geyer M. A. (2000). Animal models of deficient sensorimotor gating: what we know, what we think we know, and what we hope to know soon. *Behav. Pharmacol.* 11 185–204. 10.1097/00008877-200006000-00002 11103873

[B84] Swerdlow N. R. Braff D. L. Geyer M. A. (1999). Cross-species Studies of Sensorimotor Gating of the Startle Reflex. *Ann. N. Y. Acad. Sci.* 877 202–216. 10.1111/j.1749-6632.1999.tb09269.x 10415651

[B85] Swerdlow N. R. Geyer M. A. (1998). Using an animal model of deficient sensorimotor gating to study the pathophysiology and new treatments of schizophrenia. *Schizophr. Bull.* 24 285–301. 10.1093/oxfordjournals.schbul.a033326 9613626

[B86] Takahashi H. Hashimoto R. Iwase M. Ishii R. Kamio Y. Takeda M. (2011). Prepulse inhibition of startle response: recent advances in human studies of psychiatric disease. *Clin. Psychopharmacol. Neurosci.* 9:102. 10.9758/cpn.2011.9.3.102 23429840 PMC3569113

[B87] Takahashi H. Nakamura T. Kim J. Kikuchi H. Nakahachi T. Ishitobi M. (2018). Acoustic hyper-reactivity and negatively skewed locomotor activity in children with autism spectrum disorders: an exploratory study. *Front. Psychiatry.* 9:355. 10.3389/fpsyt.2018.00355 30127755 PMC6088201

[B88] Tobias M. R. Ito T. A. (2021). Anxiety increases sensitivity to errors and negative feedback over time. *Biol. Psychol.* 162:108092. 10.1016/j.biopsycho.2021.108092 33865907 PMC8187315

[B89] van Boxtel A. (2010). Filters for optimal smoothing of acoustic and electric blink reflex EMG responses to determine blink response magnitude. *Biol. Psychol.* 85 299–305. 10.1016/j.biopsycho.2010.07.017 20688130

[B90] van Boxtel A. Boelhouwer A. Bos A. (1998). Optimal EMG signal bandwidth and interelectrode distance for the recording of acoustic, electrocutaneous, and photic blink reflexes. *Psychophysiology* 35 690–697. 10.1111/1469-8986.3560690 9844430

[B91] Waldo M. Graze K. de Graff Bender S. Adler L. Freedman R. (1987). Premenstrual mood changes and gating of the auditory evoked potential. *Psychoneuroendocrinology* 12 35–40. 10.1016/0306-4530(87)90020-5 3588811

[B92] Wechsler D. Kodama H. (1949). *Wechsler Intelligence Scale for Children.* New York, NY: Psychological corporation.

[B93] Widmann A. Schröger E. Maess B. (2015). Digital filter design for electrophysiological data – a practical approach. *J. Neurosci. Methods* 250 34–46. 10.1016/j.jneumeth.2014.08.002 25128257

[B94] Wynn J. K. Sergi M. J. Dawson M. E. Schell A. M. Green M. F. (2005). Sensorimotor gating, orienting and social perception in schizophrenia. *Schizophr. Res.* 73 319–325. 10.1016/j.schres.2004.07.013 15653277

[B95] Yuan J. He Y. Lei Y. Yang J. Li H. (2009). Event-related potential correlates of the extraverts’ sensitivity to valence changes in positive stimuli. *Neuroreport* 20 1071–1076. 10.1097/WNR.0b013e32832e7d55 19543131

